# Geographical Distribution of *Mauremys sinensis*, *Mauremys reevesii*, and Their Hybrids in South Korea

**DOI:** 10.3390/ani14182626

**Published:** 2024-09-10

**Authors:** Hae-Jun Baek, Eujin Cheong, Youngha Kim, Kyo Soung Koo, Su-Hwan Kim, Chang-Deuk Park, Ju-Duk Yoon

**Affiliations:** 1 Invasive Alien Species Team, Bureau of Survey and Safety Research, National Institute of Ecology, Seocheon 33657, Republic of Korea; 2 National Migratory Birds Center, National Institute of Biological Resources, Incheon 22689, Republic of Korea; 3 National Ecosystem Survey Team, Bureau of Survey and Safety Research, National Institute of Ecology, Seocheon 33657, Republic of Korea; 4 Korean Environmental Geography Institute, Sejong 30141, Republic of Korea; 5 Wetland Research Team, Wetland Center, National Institute of Ecology (NIE), Changnyeong 50303, Republic of Korea; 6 Research Center for Endangered Species, National Institute of Ecology (NIE), Yeongyang 36531, Republic of Korea

**Keywords:** *Mauremys* spp., hybridization, genetic pollution, alert alien species

## Abstract

**Simple Summary:**

The global economic cost of managing invasive species exceeded $423 billion annually in 2019 and has increased at least fourfold every decade since 1970. In South Korea, the annual damage caused by invasive species is estimated to be between $93 million and $51.68 million. South Korea is vulnerable to invasive species and the cost of invasive species is estimated to be the highest among other countries, indicating the need for policy measures and responses. Most of the invasive reptiles in South Korea are introduced and released by humans. The hybridization of the Chinese striped-necked turtle *Mauremys sinensis* and Reeves’ turtle *M. reevesii* identified in the current study is also suspected to have been introduced from captivity into the wild, raising alarm bells. This suggests that the introduction of hybrids into the wild could pose a serious problem for biodiversity, especially for the *M. reevesii*, as it could undermine the genetic diversity of the population. Public awareness and education are needed to preserve South Korea’s biodiversity, and efforts must be made to reduce the number of exotic reptiles introduced into the wild.

**Abstract:**

The Chinese striped-necked turtle *Mauremys sinensis*, introduced into South Korea presumably in 2012, is considered an invasive alien species owing to its devastating impact, including hybridization with the native protected species Reeves’ turtle *M. reevesii*. Recently, the presence of *M. sinensis* has been confirmed throughout the country, and several sympatric areas with *M. reevesii* have been reported. Thus, field surveys were conducted at 47 sites across *M. sinensis* and *M. reevesii* habitats in South Korea to determine the extent of hybridization. Five sympatric sites were confirmed, and hybrid individuals were identified at four sites. Genetic analyses (*COI* and *R35*) of two individuals from Jeju Island confirmed maternal *M. reevesii* and paternal *M. sinensis* lineages. Hybridization presumably does not occur under natural conditions, and the hybrids likely originated from captive breeding. This study identifies for the first time the habitats of *M. sinensis* and its hybrids in the wild of South Korea. The management measures proposed in the current study could be of value for the conservation of the native species; however, our study did not include reproductive monitoring, and there is a need for such surveys as well as for systematic management of non-native turtles introduced into South Korea.

## 1. Introduction

Alien species in South Korea are classified as Invasive Alien Species (IAS), based on their potential for introduction, establishment and spread; their impact on ecosystems, society and the economy; and the difficulty of biological control. To date, five species of invasive alien turtles have been recorded, *Pseudemys concinna*, *Pseudemys nelsoni*, *Mauremys sinensis*, *Macrochelys temminckii*, *Chelydra serpentina*, and all species of the genus *Trachemys* [1]. The exact purpose of importation of non-native turtles is not known, but it is assumed that most are imported as pets [2,3,4,5].

One of the IAS of turtles, the Chinese striped-necked turtle *Mauremys sinensis* (order Testudines; family: Geoemydidae), is listed as “Endangered” in the IUCN Red List [6] and in CITES Appendix III [7]. This species is native to mainland China (Fujian, Guangdong, Guangxi, Hainan and Zhejiang Provinces), Taiwan and Vietnam and has been introduced to South Korea, the United States (Florida), Europe and Japan [8]. Although the specific dates and intentions of these introductions remain unconfirmed, *M. sinensis* may have been imported as an alternative to *Trachemys* spp. (red-eared sliders), which were banned from import and distribution when they were designated as IAS in 2001 [9].

*Mauremys sinensis*, a globally traded species, was identified in the wild in South Korea in 2012 [10], and its establishment in the country was confirmed in 2016 [11]. Adult *M. sinensis* individuals are approximately 25 cm long, and females grow larger than males [9]. Their recorded maximum lifespan is 22.8 years in captivity, and they are omnivorous with distinct prey preferences between males and females—adult females are omnivorous with a notable herbivorous preference, whereas adult males and young individuals are predominantly carnivorous [9]. 

Species of the Geoemydidae family frequently exhibit interspecific (and intrageneric) hybridization, which commonly occurs in captive settings. The release of such hybrids into the wild poses a substantial risk of introgression, potentially leading to the loss of unique genetic traits of wild populations. According to Stuart and Parham [12], 14 new Geoemydidae species have been reported in the past 20 years, 6 of which originated from hybridization in captivity. *Mauremys sinensis* hybridizes with congeneric species, resulting in hybrids such as *M. sinensis* × *M. reevesii*, *M. sinensis* × *M. annamensis*, and *M. sinensis* × *C. trifasciata* [13,14,15].

The semi-aquatic Reeves’ turtle *M. reevesii* is native to South Korea; it is protected by law, such as Natural Monument No. 453, and is listed as Endangered Wildlife II in South Korea [16]. Adults of the species can grow up to 30 cm in length [17]. This species can be distinguished from *M. sinensis* by its dark brown carapace with three ridges and a lemon-yellow, C-shaped patch on the side of its face. Habitat loss and destruction severely reduced the distribution of *M. reevesii*, and the recent introduction of non-native turtle species such as *M. sinensis* into its range, with potential for hybridization, has exacerbated the already precarious situation of *M. reevesii* [2,9,18,19,20,21].

There are no studies on *M. sinensis* or hybrids of *M. sinensis* and *M. reevesii* in South Korea, and there is a need to determine the distribution of these species in South Korea and the existence of hybrid individuals in the wild. This is essential for the development of effective conservation strategies for native protected species *M. reevesii*, as well as priority management points and targets for invasive species. Therefore, in the present study, we aimed to identify the current habitats of *M. sinensis* and *M. reevesii* and to establish an accurate genetic analysis method for species identification. 

## 2. Materials and Methods

### 2.1. Distribution of M. sinensis, M. reevesii and Their Hybrids 

To determine the distribution status of *M. sinensis*, *M. reevesii* and their hybrids in South Korea, a comprehensive literature review and field surveys were conducted from 2017 to 2022. Survey sites were selected based on the reports of the National Institute of Ecology, namely the “Nationwide Survey of Non-native Species in Korea from 2016 to 2021” [2,3,18,19,20] and the 2018 report “Investigating Ecological Risk of Alien Species”. 

As a result of the distribution check, 48 distribution sites of *M. sinensis* and *M. reevesii* were identified, and among them, two hybrid individuals were identified in captivity at one breeding facility located in Hwaseong, Gyeonggi-do (Table 1, No. 48). Based on the results, we conducted field surveys at 47 sites identified in the literature to confirm the outdoor distribution of *M. sinensis* and *M. reevesii* hybrids in the wild. The sites were mainly urban ecological parks and included reservoirs and rivers near schools and religious institutions. Taking advantage of freshwater turtles’ habit of basking to maintain body temperature, surveys were mainly conducted between 11 am and 4 pm. Field surveys were conducted using a pair of binoculars (SLC 8 × 42 WBHD; Swarovski, Absam, Austria) and a field scope (ATM 65HD; Swarovski). Individuals identified during field surveys were initially identified in the field based on the shape and number of lateral facial lines and the shape and color of the carapace; final species determination was made after photographical confirmation. The survey was conducted as part of the 2022–2023 project of the National Institute of Ecology ‘Monitoring of Invasive Alien Species’ project. 

### 2.2. Hybrid Specimen Sampling 

Captive surveys were conducted in 2021–2022 to collect samples for genetic analysis at seven sites (Table 1, sites 26, 38, 43–47), including four sites where hybridization had been identified through the literature and fieldwork. Turtles were captured using floating traps. The traps were made of transparent acrylic material measuring 70 cm × 70 cm, with a depth of 20 cm, and Japanese sardinella (fish) or pork was used as bait. Of the turtles collected during the survey, IAS turtles were euthanized in accordance with the Korean Biodiversity Act, hybrids (*M. sinensis* × *M. reevesii*) and *M. reevesii* were released after blood sampling, and non-study species were released without sampling.

In addition, an *M. reevesii* breeding farm in South Korea (Table 1, no. 48) was visited to collect blood samples from two individuals suspected of being subjected to hybridization in the breeding facility. Less than 2 mL blood was collected for genetic analysis from the captured individuals; *M. sinensis* and hybrids were transported to the laboratory and killed, whereas *M. reevesii* individuals were released back into the field.

A total of thirteen individuals of *M. sinensis*, *M. reevesii* and their hybrids were collected during field surveys and visits to captive breeding facility, with three individuals of *M. sinensis* (Tor019, 020, and 410), six individuals of *M. reevesii* (Tor409, 411, 412, 413, 414, and 415), and four individuals of their hybrids (Tor036, 037, 062, and 274). The survey was conducted as part of the 2022–2023 project of the National Institute of Ecology ‘Monitoring of Invasive Alien Species’ project.

### 2.3. Genetic Analysis

DNA was extracted using a Qiagen DNeasy Blood and Tissue kit (Qiagen, Hilden, Germany) following the manufacturer’s instructions. For molecular identification, the mitochondrial DNA cytochrome c oxidase subunit I (*COI*) gene and nuclear DNA *R35* gene were targeted, and novel primers were designed to amplify the target regions: the *COI* region was amplified using MAU-MT-CO1-05190f (5′-TARTTAACAGCTAAAYACCC-3′) and MAU-MT-CO1-07039r (5′-AACCTATAATYTAACCTTGACAA-3′); for sequencing, MAU-MT-CO1-05831f (5′-TAACTATCTTTTCCCTYCACCTA-3′) was used. The *R35* region of nuclear DNA intron 1 was amplified using MAU-R35-1f (5′-CAAAAGTCATTCTCTGGCTTC-3′), MAU-R35-2f (5′-GTCAGACTTCTTTGCATATTTGTAA-3′), and MAU-R35-1r (5′-CAACTATGTGCTGGACAG-3′). AccuPower PCR PreMix (BIONEER, Daejeon, Republic of Korea) was used for both amplification reactions, and 20 ng of genomic DNA was obtained from a reaction mixture of 20 µL. Polymerase chain reaction (PCR) conditions were as follows: 5 min at 95 °C for initial denaturation, 30 cycles for 20 s at 95 °C, 20 s at 55 °C, 2 min at 72 °C, and final elongation for 5 min at 72 °C. All samples were purified using an AccuPrep PCR Purification Kit (BIONEER) and sequenced on an ABI3730XL system (Applied Biosystems, Waltham, MA, USA) following the manufacturer’s instructions.

The sequences of the *COI* and *R35* fragments were edited and aligned using Geneious 5.3.6 (BIOMATTERS, Auckland, New Zealand). Multiple-sequence alignments were performed using CLUSTAL X [22]. Pairwise sequence divergence within and between *Mauremys* species was estimated using MEGA version 7 [23]. SeqPHASE (https://eeg-ebe.github.io/SeqPHASE/, accessed date: 11 August 2024) [24] was used to confirm the alleles of the parental species. 

DNA molecular phylogenies were reconstructed using the maximum likelihood method with default priors and 1000 bootstrap replicates using MEGA version 7 [24] and the best-fit model for sequence evolution was selected using the same software. For the best-fit models, 24 different nucleotide substitution models were compared and the models with the lowest BIC (Bayesian information criterion) scores were considered to best describe the substitution pattern. The best-fit model for *COI* based on the Bayesian information criterion was the T92 + G model and that for *R35* was the T92 + G + I model. To ensure accurate construction of the phylogenetic trees, 12 sequences of *M. sinensis*, 6 sequences of *M. reevesii*, 3 sequences of *Mauremys japonica*, and 5 sequences of *Mauremys mutica* were used, and *Cuora aurocapitata* (NCBI GenBank accession AY874540) and *C. amboinensis* (accession NC_014769) were used as outgroups. 

### 2.4. Compliance with Ethical Standards 

No animals were harmed, no drugs were used, and a small amount of blood was collected from a blood vessel. No animals were euthanized to collect blood samples nor was any physical harm done to the animals. Samples for molecular studies were obtained from the turtles’ tail veins. However, *M. sinensis* (IAS for South Korea) were euthanized in accordance with South Korea’s “Act on the Conservation and Use of Biological Diversity,” and part of the tail muscle was used.

## 3. Results

### 3.1. Distribution of M. sinensis, M. reevesii and Their Hybrid 

**Figure 1:**
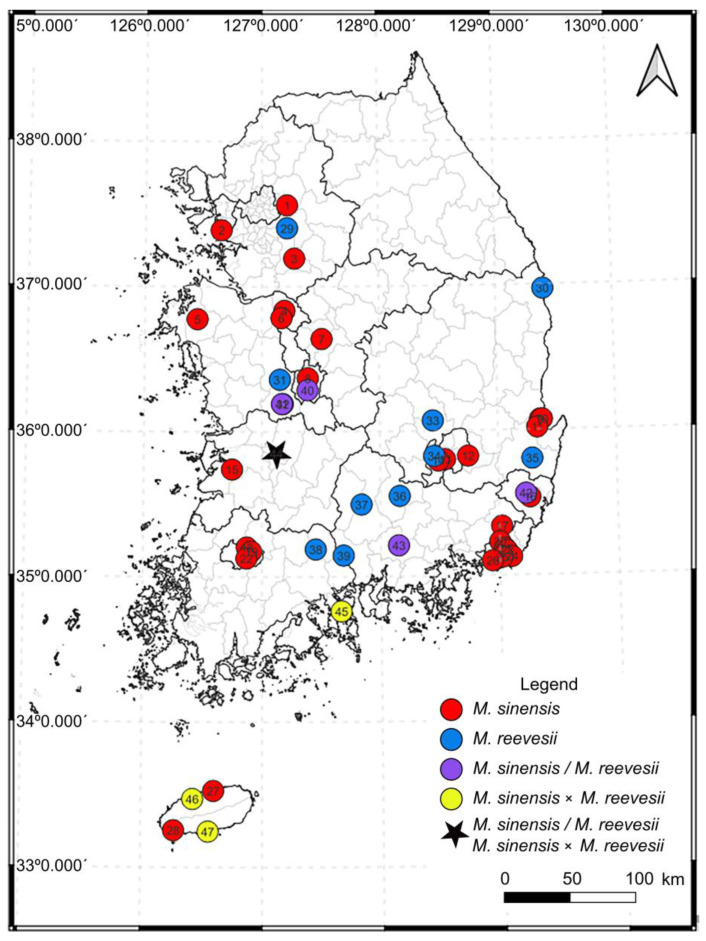
Survey sites of *Mauremys* species in South Korea and species information.

We confirmed the presence of *M. sinensis*, *M. reevesii* and their hybrid at 47 sites across South Korea (Table 1, Figure 1), and a total of 143 individuals were examined. At five of these sites, *M. sinensis* and *M. reevesii* cooccurred, and a hybrid individual occurred at four sites. The sympatric sites for the two species were Daejeon (Chimsan-dong), Nonsan (Cheonggok-ri), Ulju (Cheonsang-gil), Jinju (Jangsari) and Jeonju (Kwon Samdeok-ro), and a hybrid individual was identified in Jeonju, Yeosu, Jeju and Seogwipo (Table 1, no. 44–47). Hybridization of *M. sinensis* with *M. reevesii* was confirmed at four sites: Jeonju (Kwon Samdeok-ro), Yeosu City (Hakdong), Jeju City (Gwangnyeong-ri) and Seogwipo City (Namjung-ro). *Mauremys sinensis* was confirmed at 33 sites with 68 individuals in total, whereas *M. reevesii* occurred at only 16 sites with 66 individuals. In addition, seven hybrids of *M. sinensis* and *M. reevesii* were found in four sites. Most individuals of *M. sinensis* were observed in Busan (Choeupcheon-dong, Table 1, no. 21) and *M. reevesii* in Gurye (Gurye-eup, Table 1, no. 38). The number of individuals found by species was similar for *M. sinensis* and *M. reevesii*, but the distribution of *M. sinensis* was about twice that of *M. reevesii*, which was judged to be due to the variety of human release sites and the limited number of individuals reared by humans, rather than natural reproduction. In addition, *M. sinensis* was mainly found in urban ecological parks with high human traffic, whereas *M. reevesii* was more frequent in less-disturbed, natural habitats.

### 3.2. Occurrence of M. sinensis × M. reevesii Hybrid

**Figure 2:**
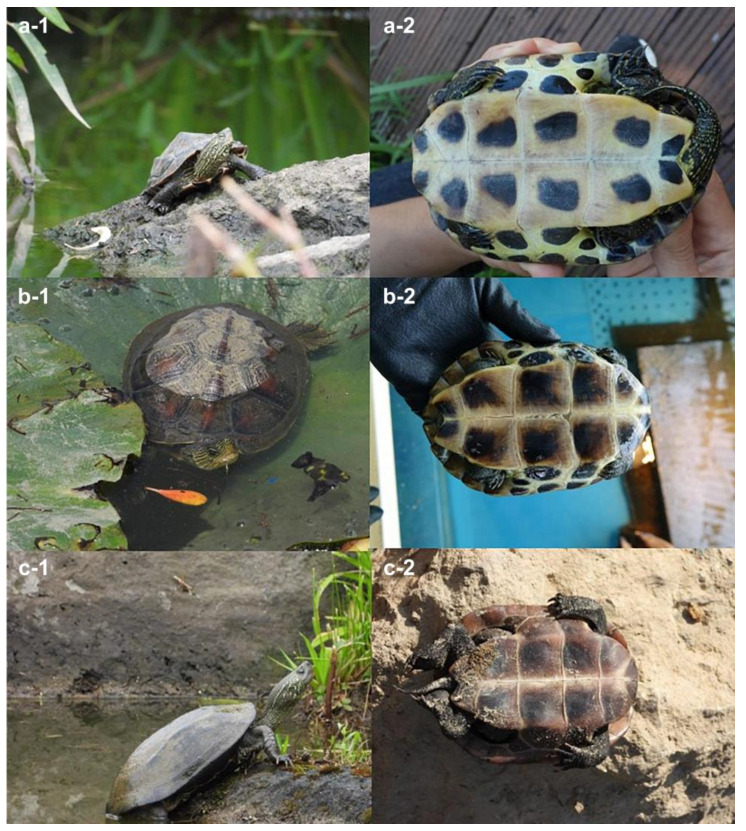
External differences among *Mauremys sinensis*, *M. reevesii* and their hybrid; (**a-1**) hybrid found on Jeju Island, (**a-2**) plastron of hybrid, (**b-1**) *M. sinensis*, (**b-2**) plastron of *M. sinensis*, (**c-1**) *M. reevesii*, and (**c-2**) plastron of *M. reevesii*.

Turtle traps were mounted at seven sites, and nine turtles were captured at four sites (Table 1, Figure 2). Five individuals of *M. reevesii* were captured in Gurye-gun (Gurye-eup, Table 1, no. 38), two individuals of *M. sinensis* were captured in Busan (Hadan-dong, Table 1, no. 26), and two hybrid individuals were captured in Jeju Island (Gwangnyeong-ro, Namseongjung-ro, Table 1, nos. 46–47), where hybridization of *M. sinensis* and *M. reevesii* was confirmed. In addition, blood samples from two hybrid individuals and one each from *M. sinensis* and *M. reevesii* were collected from the *M. reevesii* breeding farm.

### 3.3. Phylogenetic Analyses

**Figure 3:**
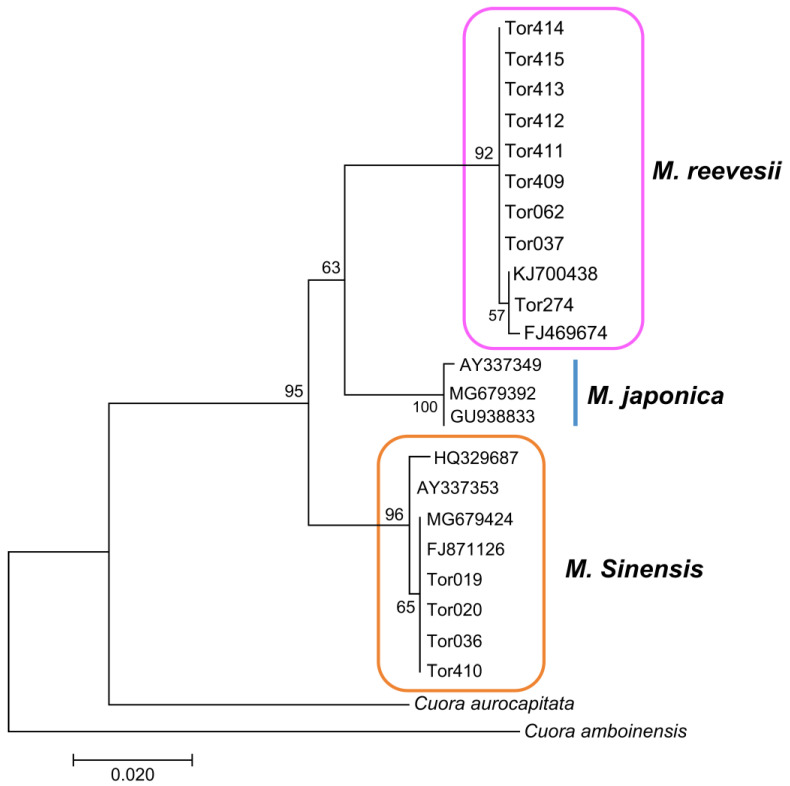
Maximum-likelihood tree based on an analysis of 13 *Mauremys* individuals using 615 bp of mitochondrial *COI* sequences, including two *Cuora* species (*C. aurocapitata* AY874540 and *C. amboinensis* NC_014769), with T92 + G and bootstrapping (1000 replications).

**Figure 4:**
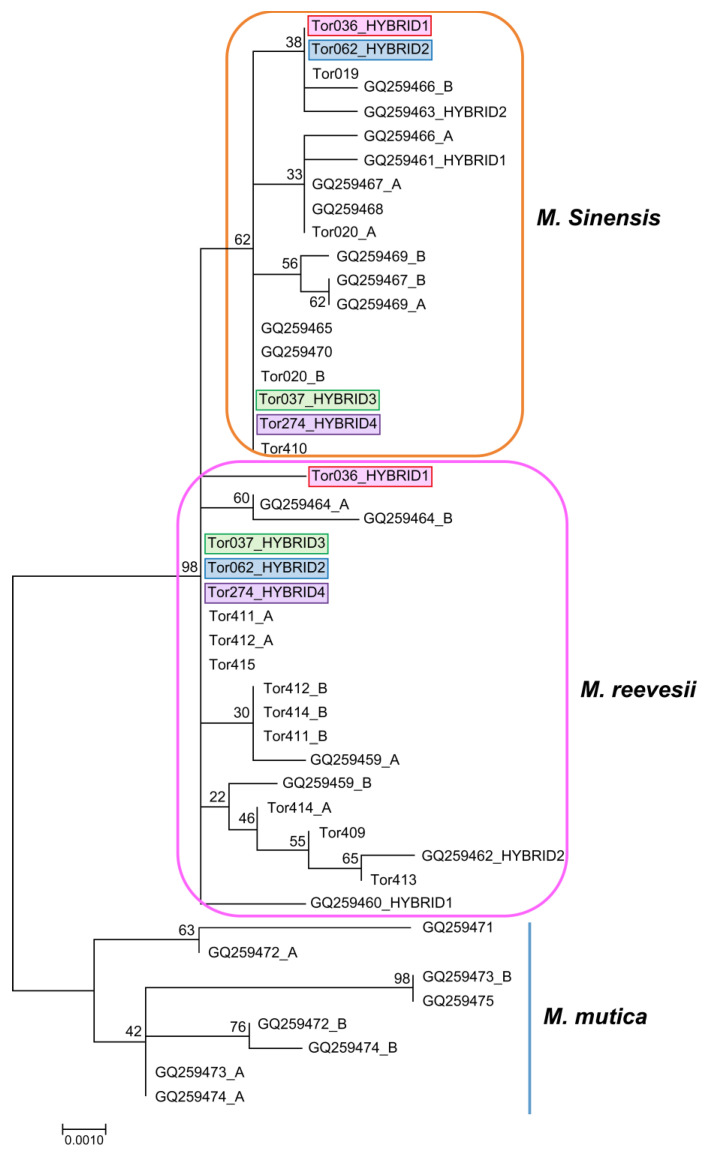
Maximum-likelihood tree based on an analysis of 13 *Mauremys* individuals using 863 bp of nuclear *R35* sequences, with T92 + I + G and bootstrapping (1000 replications).

The nine individuals captured in turtle traps and the four captive individuals were used for phylogenetic analysis. A total of 24 sequences (11 from GenBank, including sequences of 2 outgroups of the genus *Cuora*) were included for *COI* analysis and 31 sequences (18 from GenBank) were included for *R35* analysis. The partial sequence (615 bp) of mtDNA *COI* from the 13 specimens represented two distinct clades in the maximum likelihood reconstruction (Figure 3). The pairwise sequence divergence among different clades ranged from 3.6% ± 0.7% to 4.4% ± 0.8%, and the overall sequence divergence was 2.6% ± 0.4%. The four suspected hybrids (Tor 036, 037, 062, and 274) belonged to the *M. sinensis* and *M. reevesii* clades. Tor 036 belonged to *M. sinensis*, whereas as all other suspected hybrids belonged to *M. reevesii*. The 13 sequences from the same sample as *COI* comprised two separate clades: *M. sinensis* and *M. reevesii* (Figure 4). The overall sequence divergence was 0.5% ± 0.1%, and that among *Mauremys* species ranged from 0.4% ± 0.1% to 1.1% ± 0.3%. All suspected hybrid individuals possessed both *M. sinensis* and *M. reevesii* alleles, indicating that they originated from independent hybridization events. 

## 4. Discussion

In the current study, we verified the occurrence of *M. sinensis* in South Korea, its sympatric areas with *M. reevesii*, and the first record of hybridization between *M. sinensis* and *M. reevesii* in the wild. Seven hybrids of *M. reevesii* and *M. sinensis* were identified by field observation at four sites, and genetic analysis of two of the hybrids collected on Jeju Island revealed the maternal lineage as *M. reevesii* and the paternal lineage as *M. sinensis*.

To date, no breeding studies of these hybrids have been conducted in South Korea, and it is uncertain whether the hybrid individuals identified in the wild are naturally occurring or released from captivity. However, of the four sites where hybrid individuals have been identified, no individuals of *M. sinensis* or *M. reevesii* have been observed by census or collection surveys, except for Jeonju (Table 1, no. 44), and no hybrid individuals have been identified in the remaining five sites where *M. sinensis* and *M. reevesii* co-occurrence was observed, except for Jeonju, so it is likely that the hybrid individuals identified in the field to date have been released from captivity.

The only non-native turtles that have been confirmed to breed in South Korea are *Trachemys scripta*, *Pseudemys* spp. and *Chelydra serpentina*, and no other non-native species have been confirmed to breed [25,26,27,28]. In particular, no native turtle species other than *Pelodicus maackii* have been reported on Jeju Island, where the hybrid individuals were collected, and five alien turtle species (*Trachemys scripta*, *Pseudemys concinna*, *Pseudemys nelsoni*, *Mauremys sinensis* and *Podocnemis unifilis*) have been observed [3,29]. Therefore, we are certain that the hybrids identified on Jeju Island are captive-bred individuals. However, detailed breeding studies should be conducted in public areas and hybridization sites to confirm wild breeding. It is also noted that the genetic introgression of hybrids found in the wild should be investigated through more detailed surveys.

Studies on the potential effects of non-native turtles on native turtle populations [30,31,32] have confirmed overlapping ecological niches in terms of food sources and behavioral ranges. Non-native species disrupt native species diversity through competition, disease transmission and hybridization with native species. They also increase the risk of native species extinction and cause changes in ecosystem functions and service delivery [33]. Therefore, Deokjin Lake in Jeonju City, where both *M. sinensis* and *M. reevesii* and their hybrids were identified in current study, should be designated as a priority intensive management area for more aggressive removal of invasive alien turtles and habitat restoration. During the nesting season, it is recommended that nest destruction and capture surveys of turtles coming ashore should be carried out, as well as permanent capture surveys using floating traps that take advantage of the behavior of turtles basking in the sun. Park et al. [34] found that it would be beneficial to manage abandoned individuals in captivity to prevent natural release and save management costs after abandonment. Therefore, efforts such as free collection services for abandoned invasive turtles or the designation of dumping sites in urban ecological parks, where invasive turtles are commonly found, will be necessary to prevent their release into the wild.

Over the past 20 years, 161 tons of turtles have been imported into South Korea from 63 countries [1]. China, the origin of *M. sinensis*, is the largest domestic importer of turtles, with approximately 110 tons imported in the past two decades. The turtle industry in China is extensive, with as many as 10 turtle farms in Hainan alone and different species of turtles reared in the same ponds [35]. Indeed, *M. iversoni* (invalid taxon) has also been identified as a hybrid arising from co-rearing of *M. mutica* and *C. trifasciata* on turtle farms in China [35]. Genetic pollution through hybridization is common in *Mauremys* [14,15,36,37,38,39,40]. A previous study in Japan [30] found a cross-back hybrid between *M. japonica* and *M. reevesii* in the wild and reported that the fertility and hatchability of F1 individuals of *M. japonica* and *M. reevesii* were not markedly different from those of the parental species, posing a potential threat to the conservation status of *M. japonica*, which is endemic to mainland Japan. Lee et al. [41] reported that imported *M. sinensis* has caused genetic hybridization or backcrossing in approximately 57% of protected areas in China, with conservation-critical implications for native *M. reevesii* populations. As such, human-induced introductions of non-native species are emerging as a significant threat to the conservation of native species. In addition, Fong and Chen [13] also confirmed the occurrence of hybridization between *M. sinensis* and male *M. reevesii* in the wild in Taiwan, suggesting the possibility of the genetic integrity of Taiwan’s endemic tortoises being compromised by non-native species. As such, the introduction of non-native species by humans is a serious threat to the conservation of native species.

The most important aspect of establishing a basic management plan for invasive species is to identify the pathways of introduction and regulate the introduction of invasive species [42]. Of the total 2208 alien species introduced to South Korea in 2017, it was reported that about 80% of them could not be identified as to how, why or when they were introduced [43], indicating that the status of imported alien species is not clearly understood. Fortunately, the Ministry of Environment in South Korea has announced the upcoming positive list for imported tetrapods (mammals, birds, amphibians and reptiles) from December 2025. It is believed that this will allow for the management of future invasive species entering the country. However, a management plan for existing invasive species is still required. Therefore, it is necessary to establish an administrative system for the integrated management of invasive reptiles in South Korea, and an integrated organization that can conduct research on invasive species, technology development, removal management and damage assessment should be established. Studies on the management of non-native species in South Korea [1,44,45,46] have primarily focused on policies and legislation that encompass IAS; however, improving laws and policies requires time and financial resources. For rapid and more aggressive conservation, based on the results of the current study, it is recommended that the sympatric area of *M. sinensis* and *M. reevesii* should be designated and managed as priority management areas. And the sites inhabited only by *M. reevesii* (Table 1, No. 29–39) should be protected as habitat and species conservation areas. We suggest that follow-up studies should be carried out on the breeding facilities not included in this study.

## 5. Conclusions

The study was conducted to identify hybrids between *M. Sinensis* and *M. reevesii* at 47 sites throughout the country. A total of seven hybrid individuals were identified in the wild at four sites. At the sites where hybrids were found, two individuals were captured through trapping surveys, and the two individuals collected from captive breeding facilities were genetically analyzed to confirm hybridization. Hybridization may lead to the loss of the unique genotype of the Korean *M. reevesii*, and to prevent this, more active conservation measures such as nest destruction and capture of individuals using floating traps should be implemented at six sites designated as protected areas. In addition, follow-up studies such as breeding studies and genetic introgression studies should be conducted to ensure the conservation of South Korea’s biodiversity. In addition, more active public outreach and education, such as free collection of abandoned turtles and designation of dumping sites, are needed to prevent the introduction of non-native turtles into the wild.

## Figures and Tables

**Table 1:** Survey sites of *Mauremys* species in South Korea, species found, identification methods and individuals used for genetic analysis (*M. sinensis/M. reevesii* indicates sympatric distribution; *M. sinensis* × *M. reevesii* indicates hybridization observed).

No.	Locality	Species	Identification Status	Number of Individuals Observed	Id
1	Misa-dong, beon-gil, Hanam-si, Gyeonggi-do	*M. sinensis*	observation	1	
2	Haedoji-ro, Yeonsu-gu, Incheon	*M. sinensis*	observation	1	
3	Haegok-ro, Cheoin-gu, Yongin-si, Gyeonggi-do	*M. sinensis*	observation	1	
4	Anseo-dong, Dongnam-gu, Cheonan-si, Chungcheongnam-do	*M. sinensis*	observation	1	
5	Eumnae-dong, Seosan-si, Chungcheongnam-do	*M. sinensis*	observation	1	
6	Chungjeol-ro, Dongnam-gu, Cheonan-si, Chungcheongnam-do	*M. sinensis*	observation	3	
7	Yongdam-dong, Sangdang-gu, Cheongju-si, Chungcheongbuk-do	*M. sinensis*	observation	2	
8	Mannyeon-dong, Seo-gu, Daejeon	*M. sinensis*	observation	2	
9	Changpo-dong, Buk-gu, Pohang-si, Gyeongsangbuk-do	*M. sinensis*	observation	3	
10	Jangseong-dong, Buk-gu, Pohang-si, Gyeongsangbuk-do	*M. sinensis*	observation	1	
11	Daejam-dong, Nam-gu, Pohang-si, Gyeongsangbuk-do	*M. sinensis*	observation	1	
12	Gyeyang-dong, Gyeongsan-si, Gyeongsangbuk-do	*M. sinensis*	observation	2	
13	Wolgok-ro, Dalseo-gu, Daegu	*M. sinensis*	observation	1	
14	Hwawon-eup, Dalseong-gun, Daegu	*M. sinensis*	observation	2	
15	Naegi-ri, Dongjin-myeon, Buan-gun, Jeollabuk-do	*M. sinensis*	observation	1	
16	Munsu-ro, Nam-gu, Ulsan	*M. sinensis*	observation	3	
17	Beomeo-ri, Mulgeum-eup, Yangsan-si, Gyeongsangnam-do	*M. sinensis*	observation	1	
18	Yangsanje-ro, Buk-gu, Gwangju	*M. sinensis*	observation	1	
19	Yongbong-dong, Buk-gu, Gwangju	*M. sinensis*	observation	1	
20	Hwamyeong-dong, Buk-gu, Busan	*M. sinensis*	observation	2	
21	Choeupcheon-dong, Busanjin-gu, Busan	*M. sinensis*	observation	8	
22	Pungam-dong, Seo-gu, Gwangju	*M. sinensis*	observation	5	
23	Beomjeon-dong, Busanjin-gu, Busan	*M. sinensis*	observation	3	
24	UN-ro, Nam-gu, Busan	*M. sinensis*	observation	2	
25	Seodaeshin-dong-ga, Seo-gu, Busan	*M. sinensis*	observation	1	
26	Hadan-dong, Saha-gu, Busan	*M. sinensis*	observation, DNA analysis	2	Tor019, 020
27	Samyangil-dong, Jeju-si, Jeju-do	*M. sinensis*	observation	1	
28	Boseong-ri, Daejeong-eup, Seogwipo-si, Jeju-do	*M. sinensis*	observation	1	
29	Gobul-ro, Gwangju-si, Gyeonggi-do	*M. reevesii*	observation	1	
30	Susan-ri, Geunnam-myeon, Uljin-gun, Gyeongsangbuk-do	*M. reevesii*	observation	1	
31	Hadae-ri, Gyeryong-myeon, Gongju-si, Chungcheongnam-do	*M. reevesii*	observation	1	
32	Sinpung-ri, Bujeok-myeon, Nonsan-si, Chungcheongnam-do	*M. reevesii*	observation	3	
33	Yuhak-ro, Seokjeok-eup, Chilgok-gun, Gyeongsangbuk-do	*M. reevesii*	observation	1	
34	Hochon-ri, Dasan-myeon, Goryeong-gun, Gyeongsangbuk-do	*M. reevesii*	observation	4	
35	Cheongun-dong, Gyeongju-si, Gyeongsangbuk-do	*M. reevesii*	observation	1	
36	Jeongyang-ri, Daeyang-myeon, Hapcheon-gun, Gyeongsangnam-do	*M. reevesii*	observation	2	
37	Eoseo-ri, Saengcho-myeon, Sancheong-gun, Gyeongsangnam-do	*M. reevesii*	observation	1	
38	Gurye-eup, Gurye-gun, Jeollanam-do	*M. reevesii*	observation, DNA analysis	20	Tor411, 412, 413, 414, 415
39	Pyeongsa-ri, Agyang-myeon, Hadong-gun, Gyeongsangnam-do	*M. reevesii*	observation	2	
40	Chimsan-dong, Jung-gu, Daejeon	*M. sinensis / M. reevesii*	observation	*M. sinensis*: 2 *M. reevesii*: 12	
41	Chunggok-ri, Bujeok-myeon, Nonsan-si, Chungcheongnam-do	*M. sinensis / M. reevesii*	observation	*M. sinensis*: 2 *M. reevesii*: 1	
42	Cheonsang-gil, Beomseo-eup, Ulju-gun, Ulsan	*M. sinensis / M. reevesii*	observation	*M. sinensis*: 3 *M. reevesii*: 3	
43	Geumsansunhwan-ro, Geumsan-myeon, Jinju-si, Gyeongsangnam-do	*M. sinensis / M. reevesii*	observation	*M. sinensis*: 5 *M. reevesii*: 12	
44	Gwonsamdeuk-ro, Deokjin-gu, Jeonju-si, Jeollabuk-do	*M. sinensis / M. reevesii* *M. sinensis* × *M. reevesii*	observation	*M. sinensis*: 2 *M. reevesii*: 1 *M. sinensis* × *M. reevesii: 1*	
45	Hak-dong, Yeosu-si, Jeollanam-do	*M. sinensis* × *M. reevesii*	observation	*M. sinensis* × *M. reevesii: 1*	
46	Gwangnyeong-ro, Aewol-eup, Jeju-si, Jeju-do	*M. sinensis* × *M. reevesii*	observation, DNA analysis	*M. sinensis* × *M. reevesii: 1*	Tor062
47	Namseongjung-ro, Seogwipo-si, Jeju-do	*M. sinensis* × *M. reevesii*	observation, DNA analysis	*M. sinensis* × *M. reevesii: 4*	Tor274
48	In captivity Hwaseong-si, Gyeonggi-do	*M. sinensis* × *M. reevesii* *M. reevesii*, *M. sinensis*	DNA analysis	*M. reevesii* > 20 *M. sinensis*: 2 *M. sinensis* × *M. reevesii: 2*	Tor036, 037 Tor409, 410

## Data Availability

The data presented in this study are openly available in the NIE ECObank repository at https://www.nie-ecobank.kr/rdm/rsrchdoi/selectRsrchDtaDtlVw.do and https://library.me.go.kr/#/search/detail/5841308?offset=10.
